# Neural induction of porcine‐induced pluripotent stem cells and further differentiation using glioblastoma‐cultured medium

**DOI:** 10.1111/jcmm.14111

**Published:** 2019-01-04

**Authors:** Eunhye Kim, Mirae Kim, Seon‐Ung Hwang, Jongpil Kim, Gabsang Lee, Young Seok Park, Sang‐Hwan Hyun

**Affiliations:** ^1^ Institute for Stem Cell & Regenerative Medicine (ISCRM), Chungbuk National University Cheongju Chungbuk Korea; ^2^ Laboratory of Veterinary Embryology and Biotechnology (VETEMBIO) Veterinary Medical Center and Collage of Veterinary Medicine, Chungbuk National University Cheongju Chungbuk Korea; ^3^ Laboratory of Stem Cells and Cell Reprogramming, Department of Biomedical Engineering Dongguk University Seoul Korea; ^4^ Institute for Cell Engineering, Johns Hopkins University School of Medicine Baltimore Maryland; ^5^ Department of Neurosurgery, College of Medicine Chungbuk National University Hospital, Chungbuk National University Cheongju Korea

**Keywords:** glioblastoma, induced pluripotent stem cells, neural progenitor cells, pig

## Abstract

Prior to transplantation, preclinical study of safety and efficacy of neural progenitor cells (NPCs) is needed. Therefore, it is important to generate an efficient in vitro platform for neural cell differentiation in large animal models such as pigs. In this study, porcine‐induced pluripotent stem cells (iPSCs) were seeded at high cell density to a neural induction medium containing the dual Sma‐ and Mad‐related protein (SMAD) inhibitors, a TGF‐β inhibitor and BMP4 inhibitor. The dSMADi‐derived NPCs showed NPC markers such as *PLAG1*, *NESTIN* and *VIMENTIN* and higher mRNA expression of *Sox1* compared to the control. The mRNA expression of *HOXB4* was found to significantly increase in the retinoic acid‐treated group. NPCs propagated in vitro and generated neurospheres that are capable of further differentiation in neurons and glial cells. Gliobalstoma‐cultured medium including injury‐related cytokines treated porcine iPSC‐NPCs survive well in vitro and showed more neuronal marker expression compared to standard control medium. Collectively, the present study developed an efficient method for production of neural commitment of porcine iPSCs into NPCs.

## 1 INTRODUCTION

Directed neural differentiation of pluripotent stem cells (PSCs) enables researchers to generate diverse neuronal cell sources for neural development study and regenerative medicine.^1^, ^2^, ^3^ While many studies have used rodent models of neurogenesis, these models are not suitable for preclinical studies in regenerative medicine that include neural stem cell therapies or for studying human neurodegenerative diseases, because they are poor representations of the human neural system.^4^ There is therefore a need for the generation of an efficient in vitro platform for neural cell production in large animal models such as the most closely related non‐primate animal species, which is the pig.^5^ For example, pigs have similar genomic, anatomical, immunological and physiological characteristics to those of humans, as well as a comparable organ size and lifespan.^6^, ^7^, ^8^ Furthermore, porcine models are cost‐effective as they are by‐products from the abattoir, thus they are widely available compared to other large animal models. In particular, there are similarities with humans in the growth pattern of the neonatal brain and extent of peak brain growth at the time of birth.^9^, ^10^ Furthermore, pigs have gyrencephalic brain with similar grey and white matter composition and size that is more comparable to humans than are rodents.^11^ Despite these advantages, there is lack of information about efficient direct neural differentiation in porcine model.

For the robust and reproducible neuronal differentiation of PSCs, the establishment of self‐renewable neural progenitor cells (NPCs) is required.^12^, ^13^ In general, most of PSCs can differentiate into NPCs, despite significant variability in methods and efficiency for neural induction systems. Although some strategies have been used to differentiate porcine pluripotent cells into neuronal populations, little is currently known about neural development in pigs. Puy et al reported the differentiation of porcine neural cells derived from the inner cell masses (ICMs) of blastocyst‐stage embryos.^14^ Other studies have reported the derivation of NPCs from porcine isolated epiblasts from blastocysts, and evaluated their in vitro differentiation potential into neuronal and glial cells.^15^, ^16^ The majority of these initial reports have claimed that neural differentiation can be typically induced by the formation of the embryoid body (EB)^17^ or that it uses undefined factors, such as animal feeder cell lines or components.^18^ However, the EB formation method, which can form all three germ layers, produced only small numbers of neural commitment cells because of the mixed presence of other cell lineages, such as those of mesodermal or endodermal origin. Also, the inductive protocols for neuronal cells in such porcine research utilized a coculture system using a neural inducing feeder cell line called MS5. However, this is unsuitable for medical applications because the mechanism of neural induction remains unclear and animal sources pose a risk of xenogenetic pathogen transmission. This demonstrates the need to develop innovative strategies that can generate neural progenies from porcine PSCs.

Glioblastomas (GBM) are the most aggressive and malignant central nervous system (CNS) tumours.^19^ In the tumour zone, cancer cells have a remarkable ability to alter their environment for their own advantage. These cells have significant effect on the surrounding vital normal cells such as astrocytes, neurons, endothelial cells and stem cells. GBM cells are known to secrete injury‐related cytokines, including transforming growth factors (TGF‐β and TGF‐α), fibroblast growth factor (FGF), insulin‐like growth factors (IGF), platelet‐derived endothelial cell growth factor (PDGF) and vascular endothelial growth factor (VEGF).^20^ These are involved in critical tissue functions, including control of early development, and survival and differentiation of neurons and astrocytes.^21^, ^22^, ^23^ Especially, GBM is a grade IV fast‐growing glioma originated from the transformation of the normal primitive precursors of glial cells (glioblasts).^24^ Based on this aspect, the potential impact of the factors derived from GBM on maturation and differentiation of progenitor cells needs to be uncovered.

The aim of this study was thus to examine the effect of endogenous and exogenous factors in the differentiation of porcine iPSCs into NPCs. It has been known that dual small‐molecule targeting of SMAD signalling efficiently neuralizes human PSCs by blocking both BMP and Activin/Nodal pathways.^25^ By combining dual SMAD inhibitors (dSMADi) such as LDN193189 or SB431542 with the control of seeding cell density, we generated high‐yield NPCs from porcine iPSCs. The neuronal fate specification can be recapitulated in vitro using regional specific factors. In addition, the effects of brain cancer cell line‐derived conditioned medium (CM) on the differentiation of iPSC‐NPCs into neuronal‐like cells were also examined to establish an efficient system for the generation of neural cells from porcine iPSCs.

## 2 MATERIALS AND METHODS

### 2.1 Ethics statement

This study was carried out in strict accordance with the recommendations of the Guide for the Care and Use of Laboratory Animals of the National Veterinary and Quarantine Service. The protocol was approved by the Committee on the Ethics of Animal Experiments of the Chungbuk National University (permit number: CBNUA‐ 584‐13‐01). All animal subjects were killed using isoflurane anaesthesia, and all efforts were made to minimize suffering. Experimental procedures for establishing GBM cell lines were approved by the Ethics Committee and permission was granted from the institutional review board of Chungbuk National University Hospital (IRB No.: 2016‐08‐009‐002). Written informed consent was obtained for all patient samples.

### 2.2 Chemicals

Consumables were purchased from Sigma‐Aldrich Chemical Company (St. Louis, MO, USA) unless otherwise stated.

### 2.3 Culture of porcine iPSCs

The porcine iPSCs used in this study were kindly provided by Professor Jongpil Kim (Dongguk University, the Republic of Korea). The porcine iPSC lines were produced by transfecting porcine embryonic fibroblasts (PEFs) with four doxycycline‐inducible human factors (FUW‐tetO‐hOct4, FUW‐tetO‐hSox2, FUW‐tetO‐hKlf4, FUW‐tetO‐hc‐Myc and FUW‐M2rtTA from Addgene) by lentivirus. The cells were cultured in porcine ESC medium (50:50 mixture of DMEM and F10, supplemented with 15% FBS, 2 mmol/L glutamax, 0.1 mmol/L ß‐mercaptoethanol, 1x MEM non‐essential amino acids, 1x antibiotic/antimitotic) supplemented with 1000 units/ml LIF (LIF2010, Millipore) and 2 µg/ml doxycycline (D9891, Sigma) on mitotically inactivated mouse embryonic fibroblasts (MEFs) in 6‐well dishes. Cells were cultured at 38.5°C in a 5% O_2_% and 95% air atmosphere. The medium was changed daily and the cells were passaged every 3 days. They could be passaged using the 0.04% trypsin treatment and a stable morphology was maintained for more than 50 passages.

### 2.4 Neural Induction, patterning, expansion and differentiation

Porcine iPSCs were dissociated using accutase for 20 minutes, washed using porcine ESC media and pre‐plated on 1% gelatin for 15 minutes at 37°C to remove MEFs. The non‐adherent porcine iPSCs were collected after washing and replated on Matrigel‐coated dishes at a density of 40 000 or 80 000 cells/cm^2^ in MEF‐conditioned porcine ESC medium supplemented with 10 ng/ml of bFGF and 10 µmol/L Y‐27632. The porcine iPSCs were allowed to expand in cell medium for 24 hours. The initial differentiation media conditions included knockout serum replacement (KSR) media containing the dSMADi, 10 µmol/L SB431541 (TGF‐β inhibitor) and 100 nmol/L LDN 193189 (BMP4 inhibitor). On day 5 of differentiation, the dSMADi were withdrawn and N2/B27 medium, which is composed of DMEM/F12 (Gibco) supplemented with 1 × B27 (Gibco), 0.5 × N2 (Gibco), was added in increasing 25% increments (25%, 50%, 75%) every other day starting on day 4 (100% N_2_ on day 10). To initiate neuronal patterning and differentiation, we exposed porcine iPSCs treated with dSMADi to 50 ng/mL FGF8 or 0.5 µmol/L retinoic acid (RA) for 5 days with or without 500 ng/mL sonic hedgehog (SHH) in N2/B27 media. Neural expansion was performed in the presence of well‐known mitogens such as 10 ng/mL epidermal growth factor (EGF), and 4 ng/mL basic fibroblast growth factor (bFGF, Invitrogen Corporation, Carlsbad, CA).^26^, ^27^, ^28^

### 2.5 Primary culture of glioblastoma (GBM) cells

Primary human GBM cells were established from fresh specimens, and verified by routine histopathological evaluation to be GBM. The GBM specimens from human patients were removed by craniotomy and transported to the laboratory after sampled in fresh Hank's Balanced Salt Solution (HBSS, Gibco, Carlsbad, CA) in a sterile tube at 4°C, washed five times with HBSS and cut into 1 mm pieces. Following digestion with 0.1% trypsin (Gibco) for 30 minutes at 37°C, these cells were washed in HBSS and seeded at a density of 5 × 10^5^ cells/mL in 6‐well plates. Cells then were cultured in N2/B27 medium supplemented with 1% non‐essential amino acids (Gibco), 1% glutamine (Gibco), 0.1 mmol/L ß‐mercaptoethanol (Gibco) and 1% antibiotic‐antimycotic (Gibco), 10 ng/mL EGF and 4 ng/mL bFGF (Invitrogen Corporation) at a 3% O_2_ and 5% CO_2_ atmosphere. After achieving 80%‐90% confluency, the cultures were passaged at 1:3. The medium was changed twice a week. To prepare GBM‐CM, GBM cells were cultured on 100 mm culture dishes until confluent, and then the medium was replaced with 8 ml of fresh medium. After 24 hours of incubation at 37°C, the medium was collected, filtered and stored at −80°C for experiments.

### 2.6 Alkaline phosphatase (AP) activity detection

Porcine iPSCs were harvested, washed three times with PBS and fixed in 4% paraformaldehyde for 5‐7 minutes at room temperature. AP activity was then assayed with NBT/BCIP chromagen solution (Roche, Basel, Switzerland).

### 2.7 Karyotyping

To check for cytogenetic abnormalities, karyotyping of porcine iPSC or GBM cells were performed in accordance with standard cytogenetic techniques.^29^ Briefly, confluent monolayers of porcine iPSCs were treated with 10 µg/mL colcemid (Gibco, Carlsbad, CA) for 3‐5 hours to induce metaphase arrest. The cells were then gently washed three times and resuspended in a pre‐warmed hypotonic solution of 0.075 mol/L KCl (Merck, Darmstadt, Germany) and 1% sodium citrate (Merck, Darmstadt, Germany) and then incubated at 37°C for 25 minutes. Cells were then suspended by shaking the flask horizontally, transferred to a conical tube and then twice fixed at room temperature by resuspending in freshly made solution containing methane and acetic acid at 3:1 ratio (Merck). The fixed cells were subsequently added to cold wet slides, which were air‐dried, treated with trypsin (Gibco) and stained with Giemsa (GTG‐banding method). Chromosomes were then counted under a standard bright field microscopy and checked for cytogenetic abnormalities.

### 2.8 Gene expression analysis by real‐time PCR or reverse transcription PCR

The porcine iPSC‐derived NPCs or differentiated cells were analysed for the expression of neural and progenitor markers by RT‐PCR or comparative qRT‐PCR. The groups of cells were harvested separately and stored at −80°C until use. Total RNA was extracted using Trizol reagent (Invitrogen Corporation, Carlsbad, CA) according to the manufacturer's instructions. Complementary DNA (cDNA) was prepared by subjecting 0.8 μg of total RNA to reverse transcription with MMLV‐reverse transcriptase (Invitrogen Corporation) and random primers (9‐mers; Takara Bio Inc, Otsu, Shiga, Japan). For RT‐PCR, the cDNA was amplified in a 20 μL PCR reaction containing 10 pmol forward and reverse primers (iNtRON Biotechnology, SungNam, Korea), 2 units of Taq polymerase (iNtRON Biotechnology, SungNam, Korea), 10x PCR buffer (iNtRON Biotechnology, SungNam, Korea) and 5 pmol of dNTPs (iNtRON Biotechnology, SungNam, Korea). PCR reactions were performed for 35 cycles with the following conditions: denaturation for 30 seconds at 95°C, annealing for 30 seconds at 57°C and extension for 30 seconds at 72°C. The products were analysed on a 1.5% agarose gel pre‐stained with ethidium bromide with a 100‐bp ladder (iNtRON Biotechnology, SungNam, Korea) and scanned using a Gel Doc 2000 apparatus (Bio‐Rad Laboratories, Hercules, CA). To determine the conditions for logarithmic phase PCR amplification of the target mRNA, 1 μg aliquots were amplified using different number of cycles. The housekeeping genes *GAPDH* served as an internal control to rule out the possibility of RNA degradation and differences in mRNA concentration. A linear relationship was observed between gene amplification and cycle number. The 20 μL reaction mixture contained 1 U of Taq polymerase (iNtRON Biotechnology, SungNam, Korea), 2 mmol/L dNTP mix and 10 pmol of each gene‐specific primer. Alternatively, qRT‐PCR was performed with 1 μL of cDNA template, 10 μL of 2x SYBR Premix Ex Taq (Takara Bio Inc, Otsu, Shiga, Japan) and 10 pmol of each primer and carried out by 35 cycles of denaturation at 95°C for 30 seconds, annealing at 55°C for 30 seconds and extension at 72°C for 30 seconds. All oligonucleotide primer sequences are presented in Table S1. The fluorescence intensity was measured at the end of the extension phase of each cycle with threshold values set manually. Relative expression was determined by the 2^Ct^ method, with *RN18S* as a control. Experiments were repeated at least three times.

### 2.9 Immunofluorescence

Immunofluorescence (IF) was performed as follows: Cells were washed with 1x PBS containing Ca^2+^ and Mg^2+^ and fixed with 4% paraformaldehyde. The cells were washed three times with PBS and permeabilized with 0.2% Triton X‐100 for 5 minutes for intracellular markers analysis. The fixed cells were co‐incubated with blocking solution (10% goat serum in PBS) and primary antibody overnight at 4°C. The primary antibodies used in this study are listed in Table S2. The following day, cells were washed three times with washing medium (Tween‐20, Triton X‐100 and PBS) and incubated with appropriate secondary antibodies at room temperature for 1 hour. Nuclei were then stained with Hoechst 33342 and the stained cells examined using a confocal microscope and ZEN 2009 Light Edition software (Carl Zeiss, Oberkochen, Germany).

### 2.10 Statistical analysis

Statistical analysis was performed using spss 17.0 (SPSS, Inc, Chicago, IL, USA). Results are expressed as the means ± SEM. One‐way ANOVA was performed to test the null hypothesis of group differences, followed by Duncan's multiple range test or Student's *t* test. *P* < 0.05 was considered statistically significant.

## 3 RESULTS

### 3.1 Derivation of porcine NPC lines from porcine iPSCs

**Figure 1:**
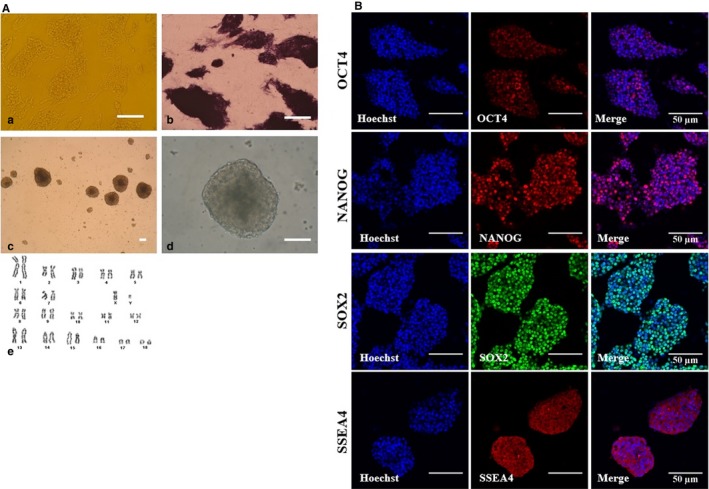
Characterization of porcine‐induced pluripotent stem cells (iPSCs). A, Representative image of porcine iPSCs with well‐defined borders (a), expression of pluripotency markers alkaline phosphatase (b) and embryoid bodies (c‐d), Scale bars = 50 A). Karyotyping results of porcine iPSCs (e). Normal chromosomes (36 + XX, p26) were observed. B, The expression of pluripotent markers in porcine iPSCs as assessed by immunofluorescence analysis. Porcine iPSCs expressed pluripotent markers, including OCT4, NANOG, SOX2 and SSEA4; Scale bars = 50 µm

To determine if porcine iPSCs used for neural induction demonstrated characteristics consistent with PSCs, we conducted a brief characterization. The porcine iPSCs showed strong AP activity and EB formation capacity with a normal karyotype (Figure 1A). Also, immunostaining revealed that porcine iPSCs were positive for the intracellular pluripotent markers OCT4, NANOG and SOX2, as well as the extracellular stem cell surface marker SSEA4 (stage‐specific embryonic antigen‐4) (Figure 1B). For inducing homogeneous neural induction and differentiation of these porcine iPSCs, we used a single‐cell‐based induction method establishing an even cell distribution. A time line showing the derivation and differentiation of porcine NPCs from this porcine iPSC line is presented in Figure S1A. Undifferentiated porcine iPSCs were dissociated into single cells and replated onto coated dishes at low and high cell seeding densities (Figure S1B) in CM supplemented with the ROCK inhibitor (Y‐2763210). After 24 hours, cells were switched from porcine iPSC conditions to knockout serum replacement (KSR) medium supplemented with two blockers of SMAD signalling, namely SB431542 (10 µmol/L) and LDN193189 (100 nmol/L), for a total of 10 days.

### 3.2 Characterization of porcine iPSC‐derived NPCs induced by dSMAD inhibition with high cell seeding density

**Figure 2:**
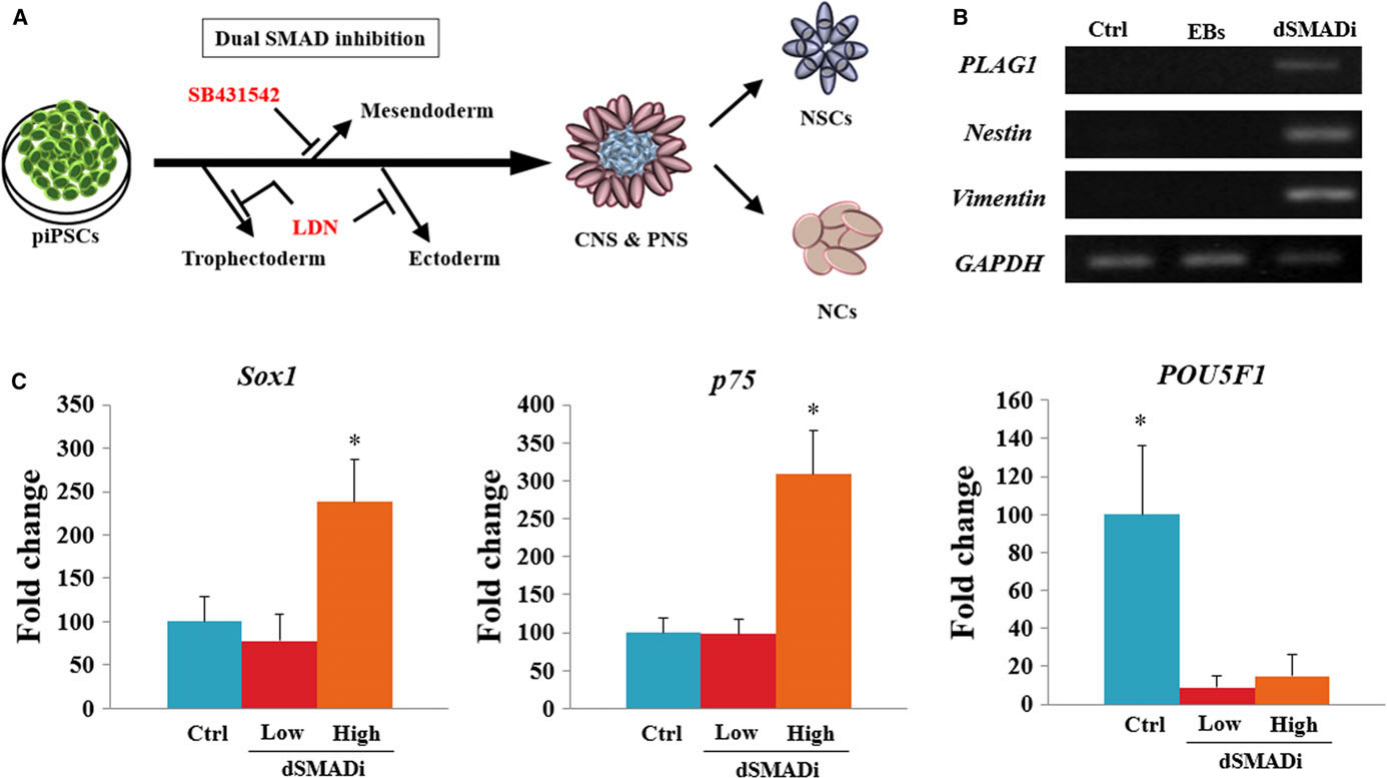
Neural induction of porcine induced pluripotent stem cells (iPSCs) using dual SMADi with different seeding cell densities. A, Scheme of neural progenitor cells (NPCs) derivation using dual SMAD inhibitors (dSMADi), SB431541 (TGF‐b inhibitor) and LDN 193189 (BMP4 inhibitor) to promote neural induction. B, Gene expression analysis of NPCs from porcine iPSCs using RT‐PCR. The experiment was replicated three times. C, mRNA expression levels (Mean ± SEM) of *SOX2*, *p75* and *POU5F1* in dual SMAD‐inhibited NPCs derived from iPSCs analysed by quantitative real‐time PCR. Within the same target mRNA, values with different superscript letters are significantly different (*P* < 0.05). The experiment was replicated three times

**Figure 3:**
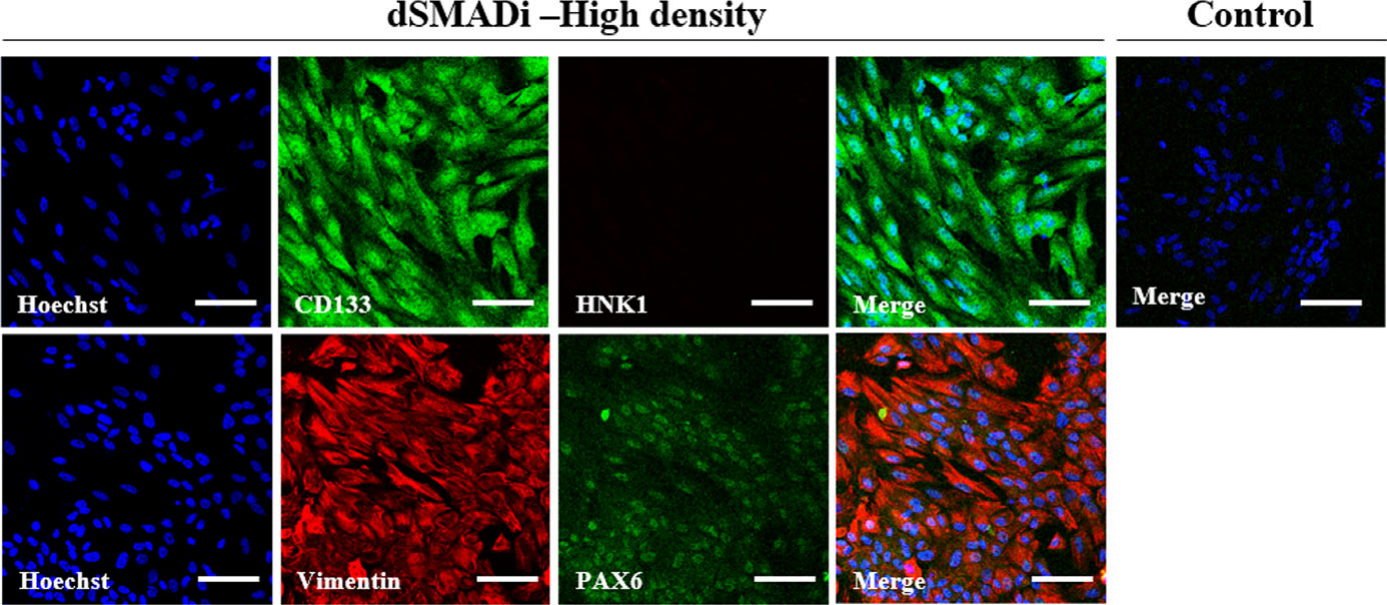
Dual SMADi‐treated porcine iPSCs with high cell density rapidly acquire a neuroectodermal phenotype within 10 d. Double‐immunofluorescence labelling of porcine iPSC‐NPCs. Green fluorescence labelling indicates CD133 or Vimentin. Red fluorescence labelling indicates HNK1 or PAX6. Nuclei are counterstained with Hoechst (blue). Scale bars = 100 µm. The experiment was replicated three times

Dual SMAD inhibition methods are known to promote neuralization of primitive ectoderm through BMP inhibition by LDN193189 and suppress mesendodermal fates by inhibiting endogenous activin and nodal signals using SB431542 (Figure 2A). Gene expression analysis confirmed that dSMADi‐treated porcine iPSCs expressed markers such as *PLAG1*, *Nestin* and *Vimentin* at day 10 (Figure 2B). There was no expression in these genes in the control group and EB group at day 10. In particular, the high cell density group revealed higher expression of the neural crest (NC) marker *p75* and neuroectodermal marker *Sox1* at day 10 of differentiation compared to those of the low‐density group and control group (Figure 2C). The expression of *POU5F1*, whose expression was maintained during pluripotent control group, decreased in both dSMADi‐treated low‐ and high‐density groups. In terms of protein levels, the expression of CNS marker, PAX6 and two early neural development markers, Vimentin and CD133, found at day 10 (Figure 3) confirmed a strong bias towards neuroectodermal lineage in the dual‐SMAD‐inhibition protocol with high cell seeding density. However, there was no expression of the NC marker, human natural killer 1 (HNK1)^30^ in the high‐density group (Figure 3). These results demonstrate that the dSMAD signalling blockade with high seeding density of porcine iPSCs promotes the differentiation of the neurodectoderm (NE).^25^ After approximately 5 days of neural induction, some of the dissociated single cells from porcine iPSC lines began to form colonies, as shown in Figure S2A. After neural induction using dSMADi, the high‐density group showed a significantly higher number of colonies than the low‐density group (0.8 ± 0.4 vs 10.2 ± 0.9, *P* < 0.01, Figure S2B).

### 3.3 Patterning and further differentiation of porcine iPSC‐NPCs

**Figure 4:**
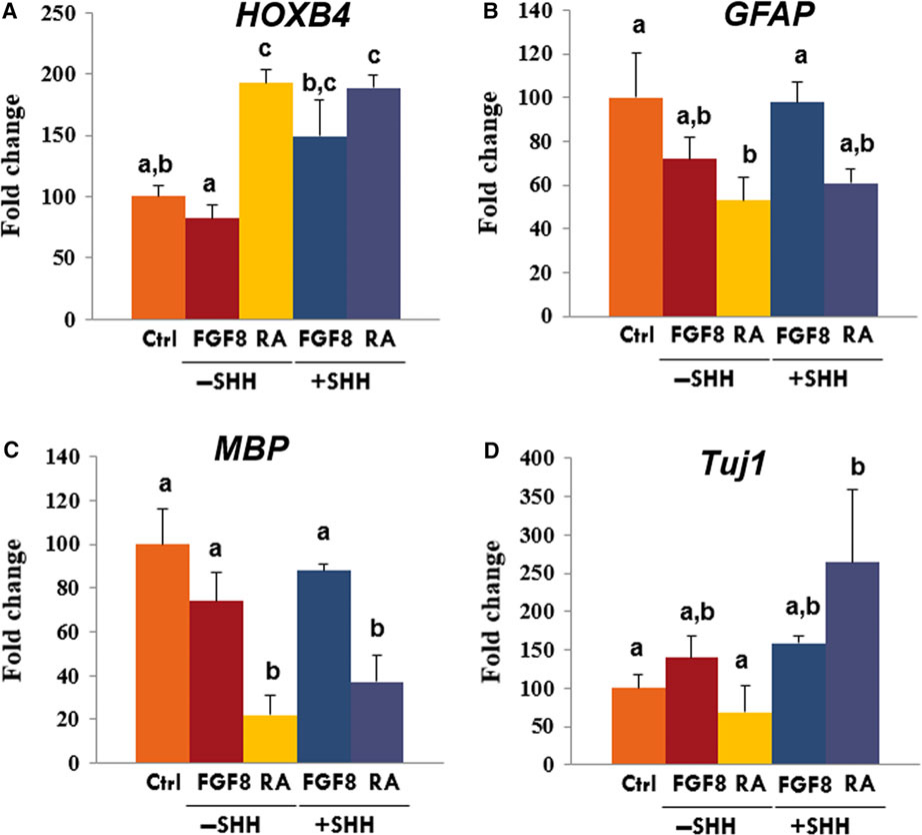
Combined treatment of FGF8, retinoic acid (RA) and SHH for patterning of iPSC‐NPCs. Comparative real‐time PCR expression of (A) HOXB4, (B) GFAP, (C) MBP and (D) Tuj1 in differentiated cells. The expression (Mean ± SEM) was measured by comparative real‐time PCR relative to the expression in non‐patterning control group. Samples were normalized to the housekeeping gene RN18S. The experiments were performed in biological triplicates. Within the same target mRNA, values with different superscript letters (a‐c) within each column indicate significant differences between groups (*P* < 0.05)

**Figure 5:**
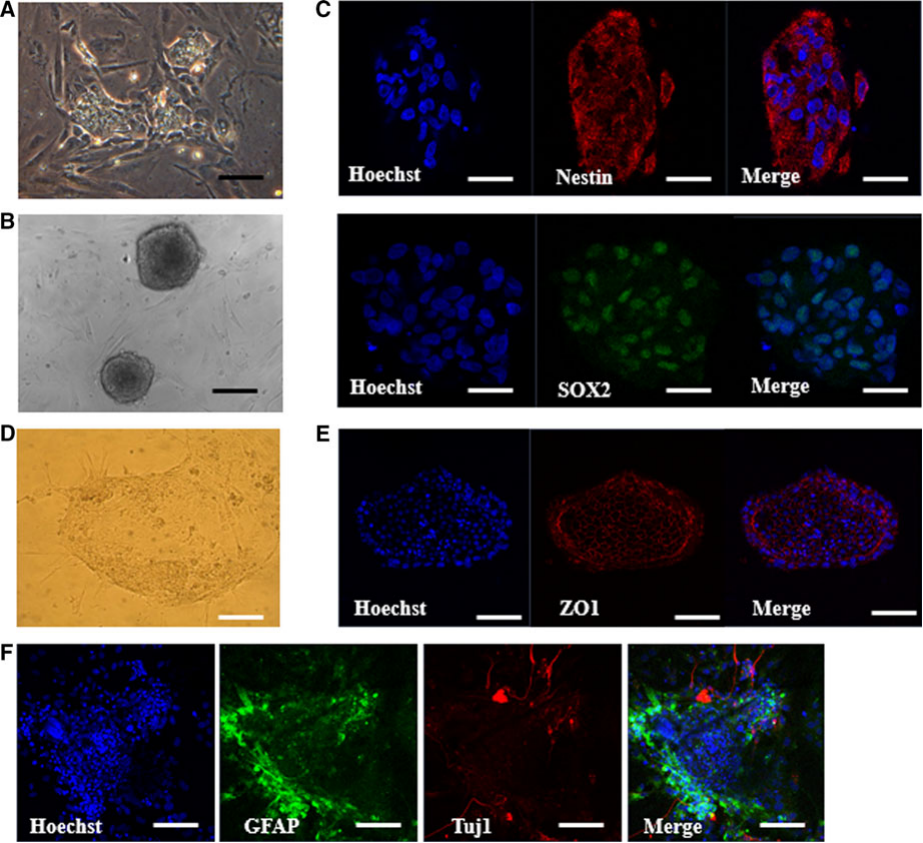
Formation of neurospheres and further differentiation from porcine iPSC‐derived NPCs. A, Representative images of neural progenitor cells (NPCs) (A) and neurosphere (B). Scale bars = 50 µm. C, Immunofluorescence labelling of the neural progenitor cell markers (NESTIN and SOX2). The experiment was replicated three times. D, Representative images of neurite outgrowth derived from clump of colonies plated on Matrigel‐coated dishes at day 2 after plating. E, Immunofluorescence labelling of the tight junction (ZO1) of the cells consist of plated clump. F, Representative immunofluorescence images that show the increased expression of the astrocyte marker GFAP in some of the cells and the outgrowths of Tuj1‐positive cells derived from the clumps. Scale bars = 50 µm. The experiment was replicated three times

To investigate whether porcine iPSC‐NPCs are responsive to instructive regionalization cues, we exposed porcine iPSCs treated with dSMADi to FGF8 or retinoic acid (RA) for 5 days with or without sonic hedgehog (SHH). Real‐time PCR analysis was performed for anterior (*OTX2*), posterior (*HOXB4*), dorsal (*PAX7*) and ventral (*NKX6.1*) markers of CNS fates. The mRNA expression of posterior marker *HOXB4* was found to significantly increase in the RA‐treated group (Figure 4). There were no significant differences in *OTX2*, *PAX7 *and *NKX6.1* (data not shown). The expression of the early neuronal marker Tuj1 was significantly up‐regulated in RA and SHH groups after following differentiation. In contrast, a myelination marker of oligodendrocytes, myelin basic protein (MBP) was significantly down‐regulated in RA with or without SHH. This result suggests that porcine iPSC‐NPCs are likely to posterior patterning in responsive to regionalization cues. Then, when cultured on ultra‐low‐attachment plates in the presence of bFGF and EGF, porcine iPSC‐derived NPCs (Figure 5A) formed neurosphere‐like aggregates (Figure 5B), which are indicative of a self‐renewal capacity.^31^, ^32^, ^33^ The cells within the spheres showed the expression of NSC marker Nestin and still the expression of Sox2 (Figure 5C). After 2 weeks, porcine NSCs differentiated into neurons positive for Tuj1 and GFAP‐positive astrocytes were also induced by LIF and CNTF conditions, whereas no expression of Nestin was found (data not shown). To further examine the neuronal differentiation potential of porcine NE cells derived by the dSMAD inhibition protocol in pigs, primary colonies derived during neural induction were mechanically dissociated into several clumps using pulled glass pipettes 10 days after culture. The clumps were then replated on Matrigel‐coated dishes and subsequent differentiated cells were examined. Two days after replating, neural progenitor‐like cells appeared and outgrowth derived from clumps of colonies showing the extensive honeycomb distribution of tight junction marker, ZO‐1 (Figure 5D,E). The outgrowth of neurite‐like cells derived from the clumps of colonies showed protein expression of a neuronal cell marker, Tuj1 at day 10 using immunofluorescence (IF) assay (Figure 5F).

### 3.4 Enhanced further differentiation of porcine iPSC‐NPCs into progeny cells using GBM‐CM

**Figure 6:**
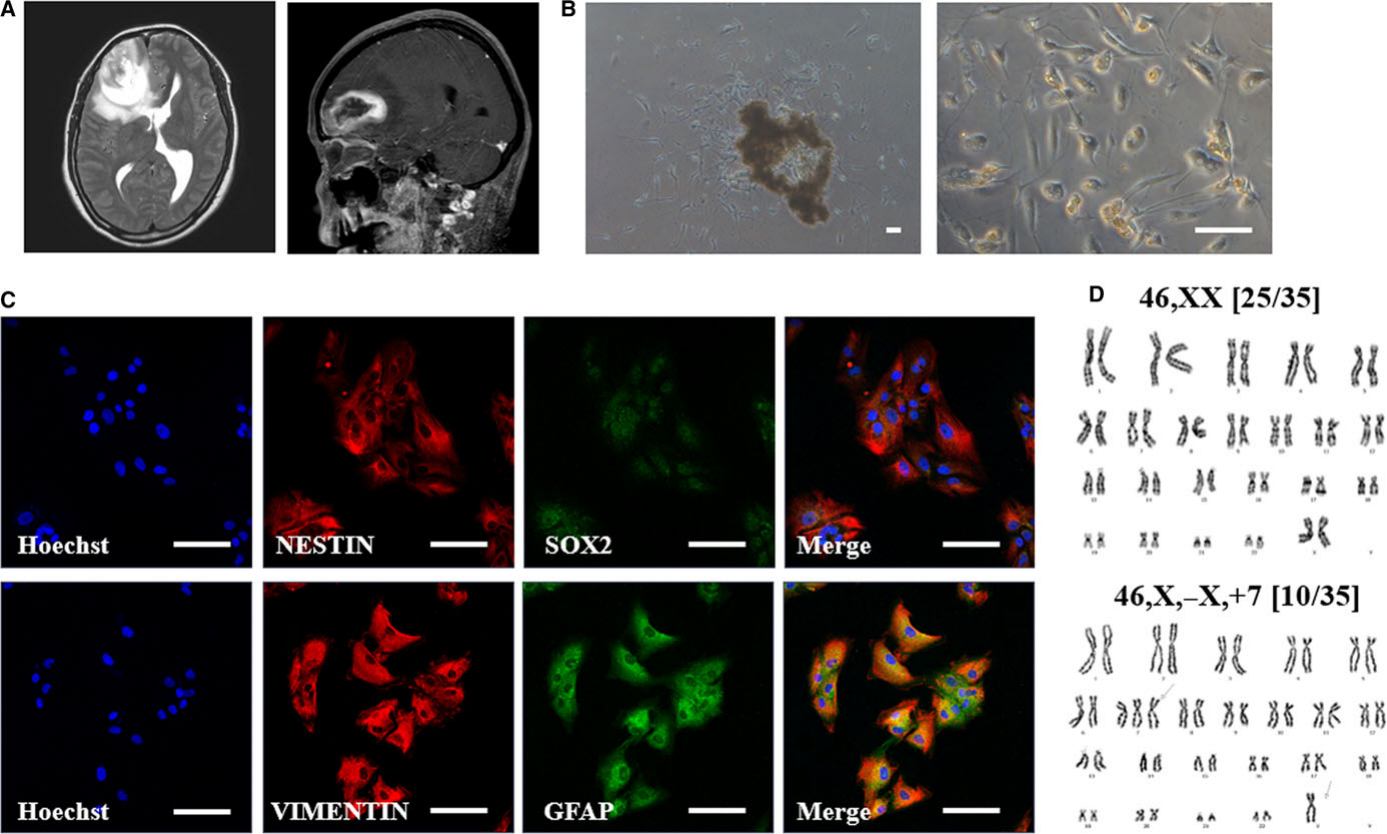
Primary culture and characterization of glioblastoma (GBM) patient‐derived cancer cell line. A, Neuroimaging of original brain tumour showed irregular shaped heterogenously enhancing intra‐axial mass in the right frontal lobe with internal haemorrhage and peritumoural oedema. B, Representative phase contrast microscopy analysis of patient‐derived primary brain tumour cells. Scale bars = 50 µm. C, Double‐immunofluorescence labelling of brain tumour cell lines. Red fluorescence labelling indicates NESTIN or VIMENTIN. Green fluorescence labelling indicates SOX2 or GFAP. Nuclei are counterstained with Hoechst (blue). Scale bars = 100 µm. D, G banded karyotype from brain cancer cell line showing chromosomal aberrations

Although we obtained porcine iPSC‐NPCs induced by dSMADi methods, they showed limited features in maturation and differentiation to neuronal progeny cells. Therefore, to obtain more robust and mature neuronal progeny cells from porcine iPSC‐NPCs, we investigated the effect of glioblastoma culture medium (GBM‐CM) on their differentiation. Neuroimaging findings using high‐resolution 3T MRI depicted an irregular‐shaped heterogenously enhancing intra‐axial mass in the right frontal lobe with internal haemorrhage and peritumoural oedema in then glioblastoma from a patient (Figure 6A). The patient‐derived GBM‐CM was primary cultured using our established protocol. The cellular morphology of the primary GBM‐derived cancer cell line was dendritic‐like (Figure 6B). IF analysis confirmed positive expression in NESTIN, SOX2, VIMENTIN and GFAP (Figure 6C). G‐banded karyotype from the primary GBM cell line revealed severe numerical chromosomal aberrations (Figure 6D). The GBM cell line showed the gain of chromosome 7 with loss of an X chromosome in 10 out of 35 metaphases (28.6%).

**Figure 7:**
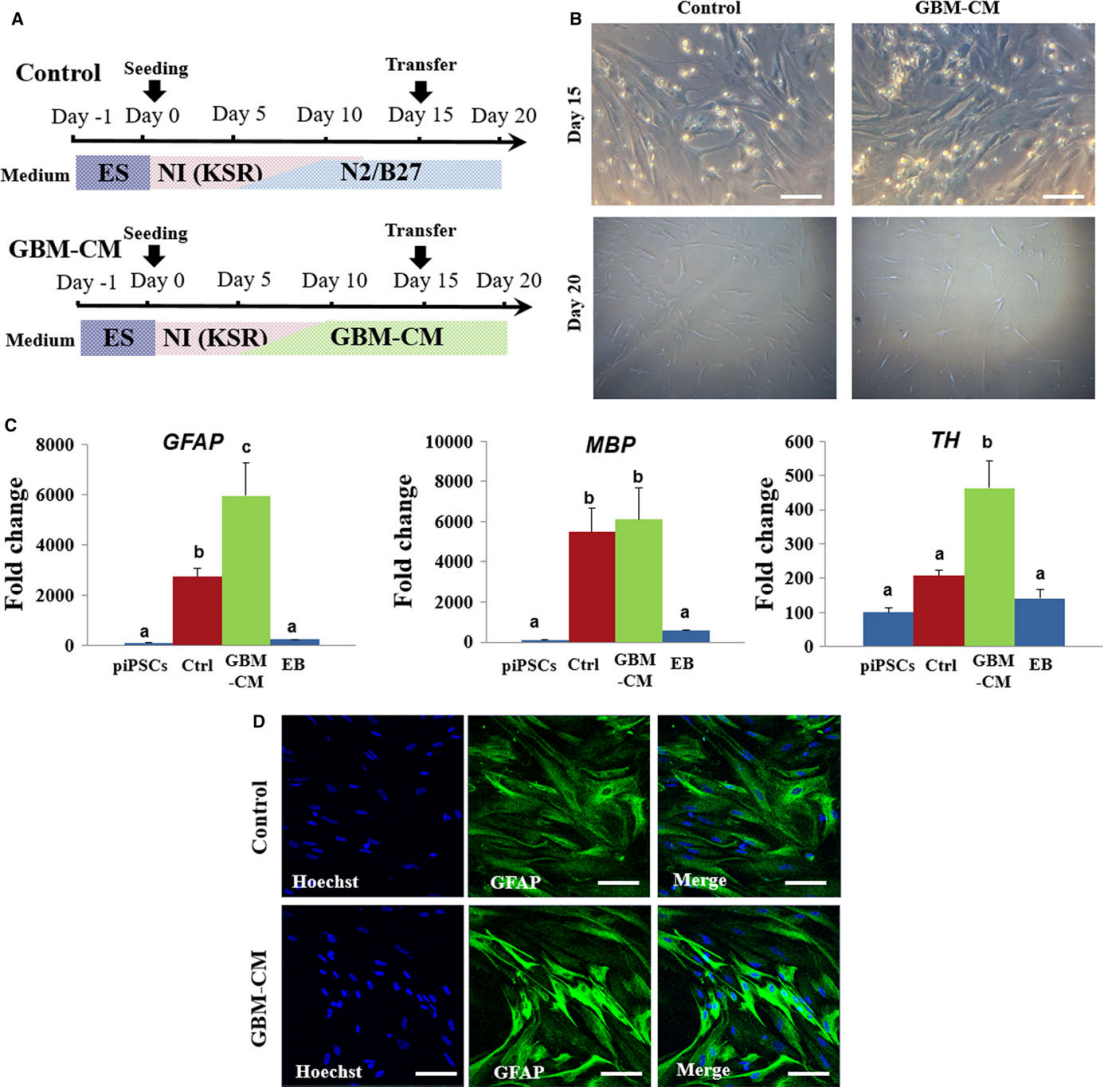
Effect of GBM‐CM on development of porcine iPSC‐derived NPCs. A, Schematic representation of differentiation protocols showing treatment of GBM‐CM during neural differentiation of porcine iPSC‐derived NPCs. B, Representative image of porcine iPSC‐derived NPCs treated with or without GBM‐CM at day 20. Scale bars = 50 µm. C, Comparative real‐time PCR expression of GFAP, MBP and TH in differentiated iPSC‐derived NPCs at day 15. The expression (Mean ± SEM) was measured by comparative real‐time PCR relative to the expression in porcine iPSCs. Samples were normalized to the housekeeping gene RN18S. The experiments were performed in biological triplicates. Within the same target mRNA, values with different superscript letters (a‐c) within each column indicate significant differences between groups (*P* < 0.05). D, Representative images of immunofluorescence that show the increased expression of the astrocyte marker GFAP in GBM‐CM treated cells. Scale bars = 50 µm

To determine whether soluble factors from GBM cells promoted the differentiation of iPSC‐NPCs, cells were treated cultured media of GBM cells instead of N2/B27 basic media (Figure 7A). GBM‐CM‐treated iPSC‐NPCs survived well in vitro when supplemented with a combination of growth factors, including EGF and bFGF (Figure 7B). The GBM‐CM‐treated differentiated cells showed an increased mRNA expression level of astrocyte marker, *GFAP*, and the dopaminergic neuron marker, tyrosine hydroxylase (TH) (Figure 7C). However, there was no significant difference in mRNA expression levels of myelination marker, MBP. In addition, GBM‐CM‐treated cells exhibited increased GFAP protein expression with a sharper morphology, which is indicative of reactive astrogliosis, compared to control group^34^, ^35^ (Figure 7D). Some of the differentiated cells progressively assumed neuronal morphological characteristics over the first 48 hours of supplement with GBM‐CM (Data not shown). Together, these data demonstrate that GBM cell‐derived soluble factors may play a pivotal role in the extensive further differentiation of porcine iPSC‐NPCs.

## 4 DISCUSSION

In this study, we developed a novel platform for neural induction and differentiation of porcine NPCs and commitment progeny cells using dSMADi and patterning factors without EB formation or feeder cell coculture. Our system also included further differentiation from porcine iPSC‐NPCs to neuronal cells using patient‐derived GBM‐CM.

Recent studies have indicated that the interactions between cells and their microenvironment play critical roles in determining cell fate in vivo.^36^, ^37^ Cells are affected by signalling factors such as soluble cytokines, cell–cell contacts and insoluble extracellular matrices.^38^ Therefore, it was thought that specific neuronal fates are determined during nervous system development through co‐ordinated integration of various endogenous and exogenous factors involved in neural differentiation. Especially, morphogens impose exogenous regional environment of neural progenitors during early stages of neural developmental.^39^ Recent studies have reported that chemically defined medium supplemented with combined small molecule compounds, dSMADi could support efficient neural induction of human PSCs.^25^, ^40^, ^41^ In particular, dSMAD inhibition yielded three times more human NPCs than did the MS5 protocol. The present results also suggest that dSMADi accelerates porcine neural developmental compared to the EB formation method. In addition, our results showed that *POU5F1* (Oct4) expression was silenced in the dSMAD inhibited group, which indicates that synergistic action of LDN 193189 and SB431542 also include destabilizing the OCT4‐mediated pluripotency network.

At the same time, the initial cell density, which is one of the endogenous factors, has an important impact in the commitment of PSCs into a particular cell lineage.^42^ It is well known that the seeding cell density can result in variable terminal cell densities, giving rise to various outcome of differentiation.^25^ In contrast to the MS5 protocol, which requires low plating density, the dSMADi method allowed for high plating densities. In the present study, the dSMAD inhibition group with low seeding density failed to initiate neural induction. We found that the initial seeded cells with high density form‐derived cells subsequently affected differentiation of the NE, the primordium of the nervous system. Therefore, the collaboration of dSMAD inhibition system and high cell density resulted in an efficient induction of early neural lineage from porcine iPSCs, which is consistent with a human study.^42^ In terms of protein levels, the nuclear expression of the early NE marker, PAX6, was observed in the high cell density dSMADi group with the expression of CD133 and Vimentin. Although the mRNA expression of placode marker, nerve growth factor receptor *p75*, was activated in the high cell seeding density group, there was no HNK1 protein, a marker for migrating NC cells at a detectable level. This indicates that neuroectodermal fate is enforced under the action of dSMAD inhibitors with high cell density under this protocol.

During embryonic developmental stages of body axis elongation, tissues express HOX genes along the rostrocaudal axis of the CNS.^43^ The spatial varied expression of HOX genes diversifies the cell fates and restricts the differentiation of specific neural progeny cells. In this study, the treatment with RA, the biologically active form of vitamin A, caused changes of the posterior gene HOX in porcine iPSC‐NPCs. Novel differentiation protocols have been reported that permit the controlled patterning towards regional‐specific types of neuronal cells by exposing the NPCs to various signalling factors^43^, ^44^; RA has been reported to be involved in anterior versus posterior patterning during CNS development.^45^ Consistent with human studies,^46^ current study showed that RA has a role in posterior neuronal specification during differentiation which indicates that the restricted regionalization of porcine iPSC‐NPCs can be converted to caudal progenitors during patterning process using a defined morphogen. After exposure to ventralizing factors such as, SHH with RA, the expression of Tuj1 was increased. It is well known that SHH affects human neural cells in ventral patterning^47^, ^48^ and generate midbrain/hindbrain dopaminergic neurons when combined with RA or FGF8 treatment.^49^ Collectively, this study highlights the remarkable possibility to reconstruct developmental specific control in vitro from porcine iPSCs. The monolayer‐based adherent differentiation protocol established in this study identifies an early role for specific signalling in neural induction and patterning of porcine iPSC‐NPCs and permits reproducible and efficient neural differentiation.

Another important finding of this study was that porcine iPSC‐NPCs led to a higher yield of differentiated neuronal progeny cells with GBM‐CM treatment. Considering the elevated levels of GFAP in GBM‐CM‐treated group with a sharper morphology, which is indicative of reactive astrogliosis, TGF‐β signalling induced by GBM‐CM might be act as an effective element in this study. The TGF‐ β govern a wide range of mechanisms in brain development, such as cell proliferation, survival and neuronal/glial differentiation.^50^ Especially, TGF‐β is thought to decline the responsiveness of astroglial cells to mitogens for proliferation and directly increase the signalling towards a differentiation pathway^51^, ^52^ The GBM‐CM encouraged differentiation or reactive responses, such as astrogliosis, which is reminiscent of the induction of repair processes such as neurogenesis and angiogenesis in mesenchymal stem cell‐transplanted damaged brain via secretion of growth and differentiation factors.^53^ Furthermore, in coincidence with the increased dopaminergic marker, TH in GBM‐CM‐treated group, TGF‐β had a neurotrophic capacity for developing dopaminergic neurons isolated from the embryonic day (E) 14 rat mesencephalon floor to varying extents^54^ These results highlight the need to develop innovative strategies based on CMs for differentiating neural progenies from porcine iPSC‐NPCs. Although further studies are needed to definitively uncover this possibility, this is the first study to report the potential application of a differentiation‐inducing effect of GBM‐CM in porcine iPSC‐NPCs.

The porcine iPSCs used in this study showed obvious expression in pluripotent markers such as POU5F1 and SSEA4, which are known to be characterized in other porcine iPSCs.^55^, ^56^, ^57^ SSEA4+ porcine iPSCs are known to be highly capable of differentiating into neural cell types similar to in vivo‐derived porcine NPCs.^58^ However, one recent study has reported that the efficiency of deriving neural rosette (NR) structure, which consists of a radial arrangement of neuroepithelial cells, may vary for each cell line depending on the expression levels of POU5F1 and SSEA4.^59^ According to this report, porcine iPSCs with low POU5F1 and high SEEA4 expression generated limited NR formation. However, we examined ZO‐1 expression of NR structure in this study regardless of the relatively low expression of POU5F1.

In conclusion, we developed a robust and efficient platform to generate porcine NPCs from porcine iPSCs and commitment protocols that differentiate into neural and glial cells. The neural induction of porcine iPSCs into NPCs was based on the chemically defined conditions such as dSMAD blockade or patterning factors and expansion and maturation using GBM‐CM. These straightforward and fast protocol developed in this study might have paramount importance for future transplantation studies in large animal models of neurodegenerative diseases. The transplantation of porcine iPSC‐NPCs may provide details on their regenerative potential in vivo.

## CONFLICT OF INTEREST

The authors declare no competing interests.

## AUTHOR CONTRIBUTIONS

EK, MK, SUH and SHH designed research; EK performed the experiments, analysed the data, drafted and revised the manuscript. JK, GL, YSP and SHH supervised the design of the study and revised the manuscript. All authors critically reviewed the manuscript for intellectual content and gave final approval for the version to be published.
