# Supplementary figures and images for: Neural induction of porcine‐induced pluripotent stem cells and further differentiation using glioblastoma‐cultured medium
Source: J Cell Mol Med. 2019 Jan 4;23(3):2052–63. doi: 10.1111/jcmm.14111 (PMC6378232; doi:10.1111/jcmm.14111)

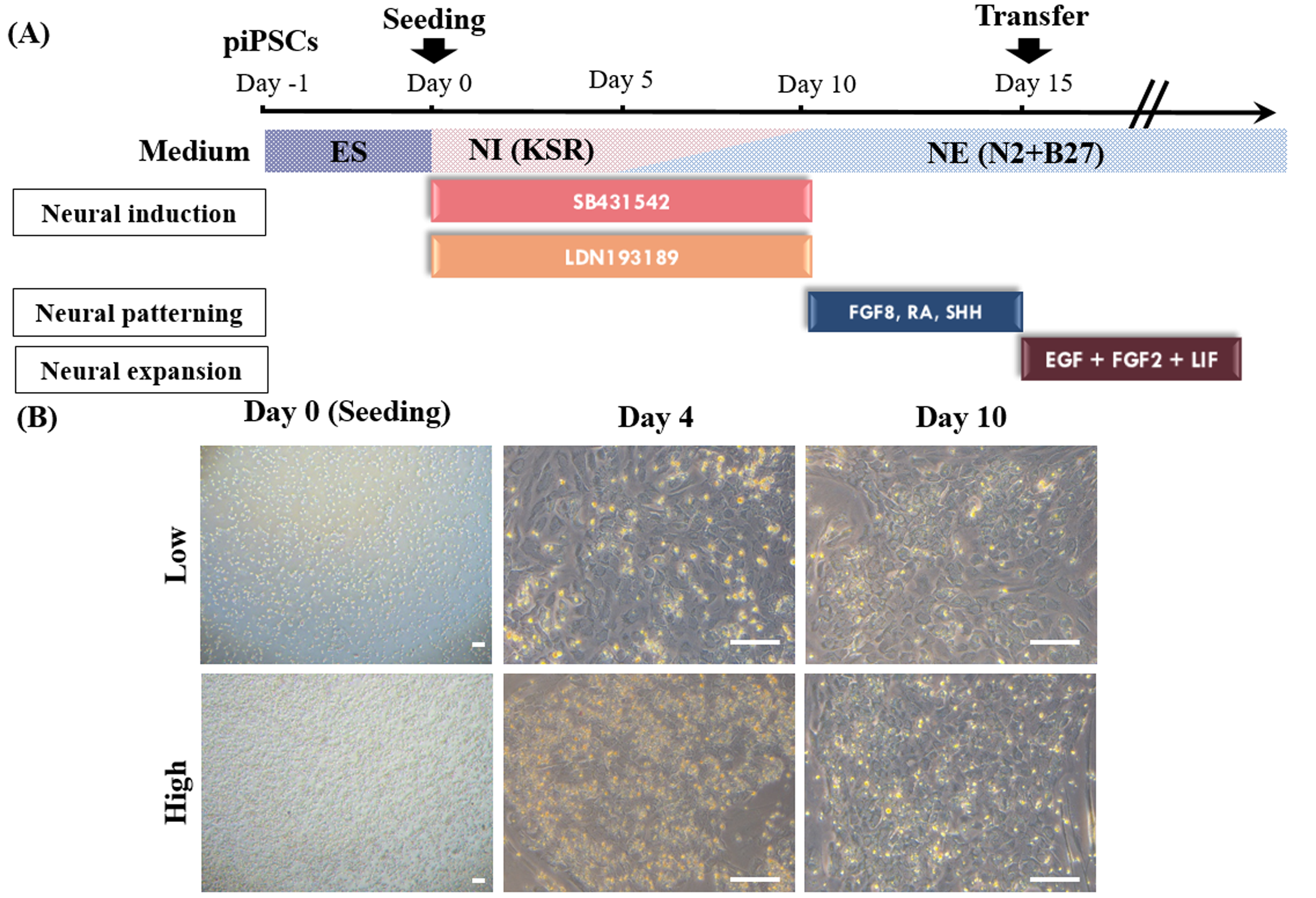

Supplement: Supplementary file 1 [file JCMM-23-2052-s001.tif]

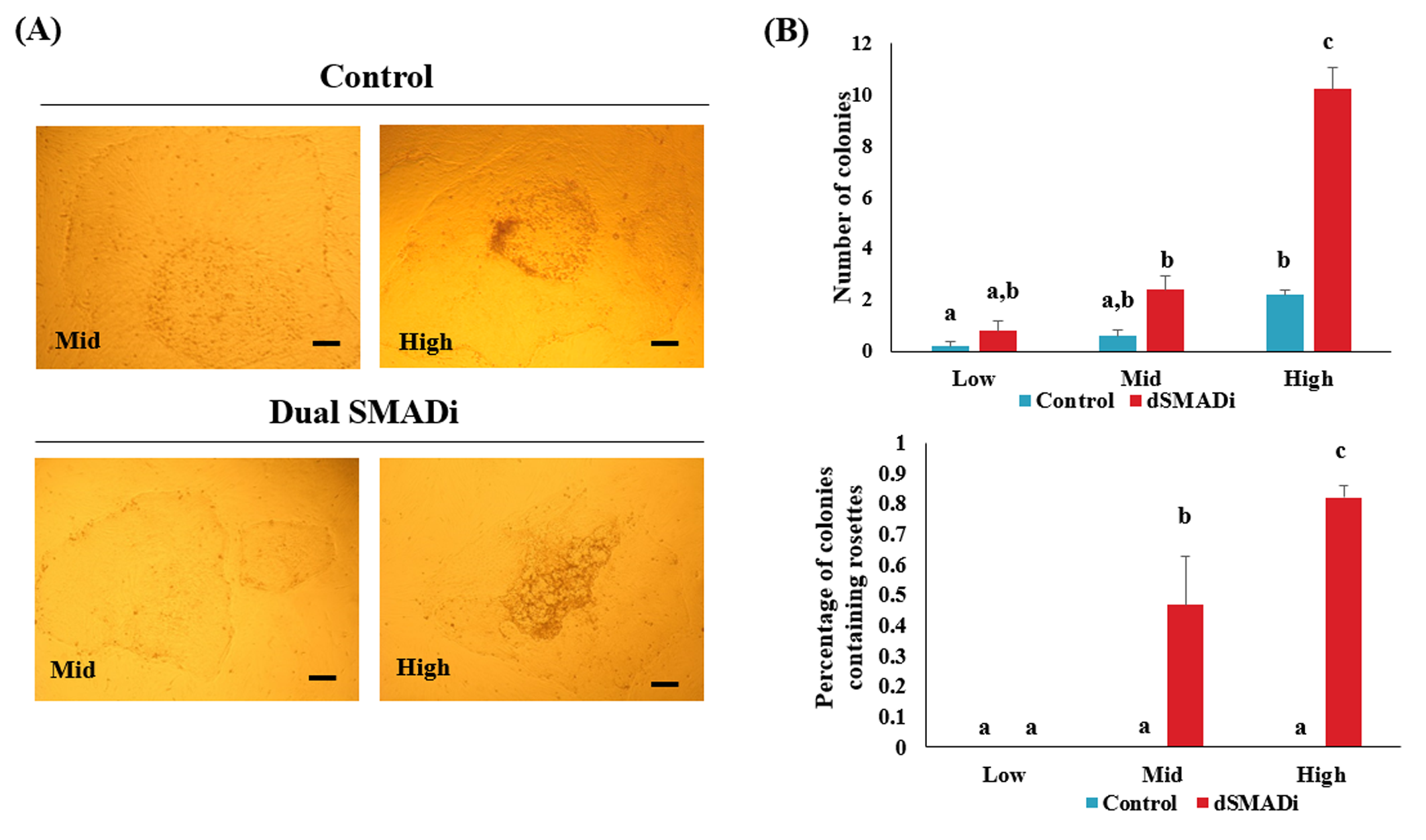

Supplement: Supplementary file 2 [file JCMM-23-2052-s002.tif]
