# Supplementary material for: Neural induction of porcine‐induced pluripotent stem cells and further differentiation using glioblastoma‐cultured medium
Source: J Cell Mol Med. 2019 Jan 4;23(3):2052–63. doi: 10.1111/jcmm.14111 (PMC6378232; doi:10.1111/jcmm.14111)
Supplement: Supplementary file 3 [file JCMM-23-2052-s003.docx]

**Supplement Figure 1. Neural differentiation of porcine iPSCs.** (A) Schematic representation of differentiation protocols showing treatment duration for neural induction, patterning, and expansion. (B) Representative morphological changes during neural induction in different cell densities. Cell densities are shown here for comparison. (Low; 40,000 cells/cm^2^, High; 80,000 cells/cm^2^) when single cell seeding. Scale bars = 50 µm.

**Supplement Figure 2. Formation of colonies during neural induction.** (A) Representative images of colonies with or without dSMADi treatment, Scale bars = 100 µm (B) Number of colonies and percentage of colonies containing rosettes with or without dSMADi treatment under different seeding cell densities. Values with different superscript letters (a-c) within a column differ significantly (P < 0.05). Experiment was repeated four times.
