# Supplementary material for: Neural induction of porcine‐induced pluripotent stem cells and further differentiation using glioblastoma‐cultured medium
Source: J Cell Mol Med. 2019 Jan 4;23(3):2052–63. doi: 10.1111/jcmm.14111 (PMC6378232; doi:10.1111/jcmm.14111)
Supplement: Supplementary file 4 [file JCMM-23-2052-s004.docx]

**Supplement table 1. Primers used for gene expression analysis**

| Genes | Primer sequences | Products |
| --- | --- | --- |
|  |  | size (bp) |
| *POU5F1* | F: 5’-AACGATCAAGCAGTGACTATTCG-3’ | 153 |
|  | R: 5’-GAGTACAGGGTGGTGAAGTGAGG-3’ |  |
| *SOX1* | F: 5’-CACAACTCGGAGATCAGCAA-3’ | 173 |
|  | R: 5’-GTCCTTCTTGAGCAGCGTCT-3’ |  |
| *P75 NTR* | F: 5’-GTGGAGATGGAGATGATATGGAA-3’ | 338 |
|  | R: 5’-GAAGGCAATCTCCAATTAGAAGC-3’ |  |
| *PLAG1* | F: 5’-TGGGGAATCTCTGCCCCATA-3’ | 529 |
|  | R: 5’-CCTTACTAGGCAGTGCCACA-3’ |  |
| *Nestin* | F: 5’-GGCAGTGGTTCCAAGGCT-3’ | 162 |
|  | R: 5’-GGCTGGCATAGGTGTGTCAA-3’ |  |
| *Vimentin* | F: 5’-GTGATGTCCGCCAGCAGT-3’ | 218 |
|  | R: 5’-GCGTTCCAGAGACTCGTT-3’ |  |
| *HOXB4* | F: 5’-TCACGTGAGCACGGTAAAC -3’ | 234 |
|  | R: 5’-TGTTGGGCAACTTGTGGTCT -3’ |  |
| *Tuj1* | F: 5’-GTGGTGCGGAAGGAGTGTG -3’ | 218 |
|  | R: 5’-TGGTGGATGGACAGCGTGG -3’ |  |
| *GFAP* | F: 5’-TTGACCTGCGACGTGGAGTC-3’ | 225 |
|  | R: 5’-AGGTGGCGATCTCGATGTCC -3’ |  |
| *MBP* | F: 5’-GAGGCAGAGCTCCTGACTACAAA-3’ | 101 |
|  | R: 5’-GTCCCGTCCTCCCAGCTT -3’ |  |
| *TH* | F: 5’-GCACGCCTCCTCGCCCAT-3’ | 181 |
|  | R: 5’-CTCCACCGTGAACCAGTA -3’ |  |
| *GAPDH* | F: 5’-GTCGGTTGTGGATCTGACCT-3’ | 207 |
|  | R: 5’-TTGACGAAGTGGTCGTTGAG-3’ |  |
| *RN18S* | F: 5’-CGCGGTTCTATTTTGTTGGT-3’ | 219 |
|  | R: 5’-AGTCGGCATCGTTTATGGTC-3’ |  |

F: Forward, R: Reverse

**Supplement table 2. Antibodies used for immunofluorescence staining**

| Detection of | Name | Host species | Dilution | Manufacturer |
| --- | --- | --- | --- | --- |
| ESC^1^ | OCT4 | Rabbit IgG | 1:200 | Abcam AB19857 |
| ESC^1^ / NPC^2^ | NANOG | Rabbit IgG | 1:200 | Abcam AB70482 |
| ESC^1^ / NPC^2^ | SOX2 | Rabbit IgG | 1:200 | Millipore AB5603 |
| ESC^1^ | SSEA4 | Mouse IgG | 1:200 | Abcam AB16287 |
| NPC^2^ | CD133 | Rabbit IgG | 1:200 | Proteintech 18470-1-AP |
| NC^3^ | HNK1 | Mouse IgM | 1:200 | Sigma C0678 |
| NPC^2^ | VIMENTIN | Mouse IgG | 1:200 | Invitrogen MA5-11883 |
| NE^4^ | PAX6 | Rabbit IgG | 1:200 | Millipore AB2237 |
| NPC^2^ | NESTIN | Mouse IgG | 1:500 | Millipore MAB5326 |
| Astrocytes / Radial glia | GFAP | Rabbit IgG | 1:1000 | Millipore PAB5804 |
| Young neurons | TUJI | Mouse IgG | 1:200 | Millipore MAB1637 |

^1^Embryonic stem cell, ^2^Neural progenitor cell, ^3^Neural crest, ^4^Neuroectoderm
